# 氟马替尼联合多药化疗治疗Ph^+^急性淋巴细胞白血病12例疗效及安全性分析

**DOI:** 10.3760/cma.j.issn.0253-2727.2021.10.011

**Published:** 2021-10

**Authors:** 瑞华 米, 琳 陈, 海平 杨, 秀丽 魏, 佳 刘, 青松 尹, 丽娜 张, 旭东 魏

**Affiliations:** 1 郑州大学附属肿瘤医院、河南省肿瘤医院 450008 The Affiliated Cancer Hospital of Zhengzhou University/Henan Cancer Hospital, Zhengzhou 450008, China; 2 河南科技大学第一附属医院，洛阳 471003 The First Affiliated Hospital, and College of Clinical Medicine of Henan University of Science and Technology, Luoyang 471003, China; 3 新乡市第一人民医院 453000 The First People's Hospital of Xinxiang City, Xinxiang 453000, China

Ph阳性急性淋巴细胞白血病（Ph^+^ ALL）是ALL的一种特殊类型，其预后不佳，酪氨酸激酶抑制剂（TKI）极大改善了该类患者预后[1]。二代TKI尼洛替尼与达沙替尼因具有更强的抗BCR-ABL及ABL激酶区突变能力而应用于慢性髓性白血病（CML）及Ph^+^ ALL的治疗。刘莹等[2]和杨飞等[3]报道，一线应用二代TKI治疗Ph^+^ ALL长期疗效优于一代TKI。氟马替尼为我国自主研发的二代TKI，目前未见其治疗Ph^+^ ALL患者的报道，现将我们应用氟马替尼联合多药化疗治疗12例Ph^+^ ALL患者的疗效及安全性报道如下。

## 病例与方法

1. 病例：以2015年7月至2020年12月在郑州大学附属肿瘤医院、河南科技大学第一附属医院和新乡市第一人民医院住院的新诊断的或复发的或既往曾接受过伊马替尼、达沙替尼出现耐药或不耐受而接受氟马替尼治疗的Ph^+^ ALL患者12例为研究对象。入组标准：美国东部肿瘤协作组（ECOG）评分<2分，重要脏器功能良好，患者具体临床资料见表1。

**表1:** 12例Ph^+^急性淋巴细胞白血病患者初诊时的临床资料

例号	性别	年龄（岁）	WBC (×10^9^/L)	PLT (×10^9^/L)	骨髓象	染色体核型	BCR-ABL基因类型	BCR-ABL/ABL值(%)	基因突变	ABL激酶区突变位点
1	男	31	43.2	28	增生明显活跃,原始幼稚淋巴细胞92.4%	45, XY, del(6)(q21), t(9;22)(q34;q11),−12[6]	P210	87.80	PAX5、SH2B3、IL7R	−
2	女	57	3.5	9	增生极度活跃,原始幼稚淋巴细胞48.0%	46, XX, t(9;22)(q34;11.2q)[10]	P210	162.63	SETD2、SH2B3	−
3	男	33	136.9	27	增生极度活跃,原始幼稚细胞96.0%	46, XY, t(9;22)(q34;q11,2)[7]/46, XY[3]	P190	100.00	−	−
4	女	59	16.3	32	增生明显活跃,幼稚淋巴细胞62.0%	46, XX, t(9;22)(q34;q11,2)[10]	P190	140.64	−	−
5	男	60	17.0	14	增生明显活跃,幼稚淋巴细胞86.0%	47, XY, t(9;22)(q34;q11.2), +mar[2]/46, XY[3]	P210	143.51	−	−
6	男	33	16.8	98	增生极度活跃,幼稚淋巴细胞90.5%	46, XY, t(9;22)(q34;q11.2)[3]	P190	93.20	ATM	−
7	男	41	139.1	56	增生极度活跃,原始幼稚淋巴细胞88.4%	46, XY, t(9;22)(q34;q11.2)[1]/[9]46, XY[1]	P210	152.75	−	−
8	男	57	89.0	150	增生明显活跃,原始幼稚淋巴细胞79.3%	46, XY, t(9;22)(q34;q11.2)[15]	P210	68.50	TET2	−
9	女	58	28.8	26	增生明显活跃,原始幼稚淋巴细胞83.0%	45-46, XX, t(9;22)(q34;q11), +der(9)t(9;22),+16,−22, der(22)t(9;22)[5]	P210	75.00	−	−
10	男	46	35.0	67	增生明显活跃,原始幼稚淋巴细胞52.7%	46, XY[10]	P190	79.80	−	−
11	男	62	20.6	68	增生明显活跃,原始幼稚淋巴细胞79.2%	46, XY, t(9;22)(q34;q11.2)[10]	P190	83.40	−	−
12	男	47	69.8	39	增生极度活跃,原始幼稚淋巴细胞92.2%	46, XY, t(9;22)(q34;q11.2)[5]/46, XY[5]	P210	197.65	−	−

注：−：阴性

2. 诊断依据：诊断符合文献[4]标准，根据骨髓细胞形态学、免疫表型分析、细胞遗传学、分子生物学（MICM）进行诊断分型，排除CML急淋变患者。

3. 染色体核型分析：按常规制备染色体，R显带，Giemsa染色后进行核型分析。异常核型根据《人类细胞基因组学国际命名体系（ISCN2016）》进行描述。剩余细胞悬液置−20 °C保存备用。

4. 融合基因检测：采用RT-PCR技术检测58种白血病相关融合基因的异常情况。若检测到BCR-ABL基因阳性，后采用RQ-PCR法确定BCR-ABL融合基因拷贝数，并通过一代或二代测序检测ABL激酶区突变。

5. 高通量测序靶向检测基因突变：使用PCR引物扩增目的基因热点区域（108种白血病相关基因），采用Ion Torrent PGM测序平台（美国ThermoFisher公司产品）进行测序。测序后数据利用人基因组数据库（HG19）、COSMIC、1000 genomes和dbSNP等数据库进行分析。平均基因覆盖率大于99％，平均测序深度为1 500×，目标区域测序深度超过1 000×，检测灵敏度5％。

6. 10色流式细胞术（FCM）检测微小残留病（MRD）水平：采集患者骨髓3 ml，肝素抗凝，于2 h内上流式细胞仪检测。FCM获取的细胞数为>500 000个。以白血病细胞占骨髓有核细胞总数的比例作为MRD值，以MRD≥0.01％为阳性，MRD<0.01％为阴性。

7. 治疗方案：诱导化疗均以VP方案为基础（长春新碱+泼尼松±柔红霉素±环磷酰胺±左旋门冬酰胺酶或培门冬酶），强化治疗给予Hyper-CVAD/MA（A/B）、CAM（环磷酰胺+阿糖胞苷+6-巯嘌呤）、大剂量甲氨蝶呤（MTX）、COAD（环磷酰胺+长春新碱+阿糖胞苷+地塞米松）等方案序贯巩固治疗。部分患者治疗过程中行异基因造血干细胞移植（allo-HSCT）。治疗期间采用MTX、地塞米松及阿糖胞苷三联鞘内注射预防中枢神经系统白血病。

8. TKI使用情况：一经确诊，推荐所有患者尽早应用TKI。TKI剂量：伊马替尼400～600 mg/d，达沙替尼100～140 mg/d，氟马替尼600 mg/d，巩固及维持治疗期间持续口服TKI，allo-HSCT后1～2年内行TKI维持治疗，本研究入组患者均未应用尼洛替尼。

9. 安全性评估：依据WHO制定的不良反应评价标准将患者不良反应级别分为0～Ⅳ度，观察氟马替尼的主要不良反应，包括水肿、四肢疼痛、皮疹、腹泻和肝肾功能及心电图情况等。

10.疗效评价标准及随访：形态学完全缓解（CR）与复发的定义参照文献[4]。主要分子学反应（MMR）定义为BCR-ABL^IS^≤0.1％（ABL转录本>10 000）。完全分子学反应（CMR）指在可扩增ABL转录本水平下无法检测到BCR-ABL转录本。随访截止日期为2020年12月31日。无进展生存（PFS）时间指患者开始接受氟马替尼治疗到出现任何原因引起的治疗失败的时间，包括复发、BCR-ABL水平升高、死亡或疾病进展等；总生存（OS）时间指患者接受氟马替尼治疗开始至随访截止的时间，死亡患者则计算至死亡日。

## 结果

1. 临床特征：12例Ph^+^ ALL患者，男9例，女3例，中位年龄52（31～62）岁。BCR-ABL融合基因类型：5例为P190阳性，7例为P210阳性。疾病状态：初治7例，复发2例，CR但BCR-ABL融合基因进行性升高2例，CMR但对达沙替尼不耐受1例。氟马替尼治疗前ABL激酶区突变位点除例5和例10分别检出T315I和F317I突变外，其余均是阴性。12例患者二代测序共检出6种伴随基因突变，分别为PAX5、SH2B3、IL7R、SETD2、ATM、TET2。

2. 治疗情况与临床疗效：初治和复发患者共9例，均在诱导治疗期即开始给予氟马替尼治疗，治疗28 d时9例（100％）患者获得形态学CR；4例（44.4％）患者获CMR（其中例5的T315I突变消失），2例（22.2％）患者获MMR，3例融合基因水平未达到MMR；8例患者FCM MRD阴性，1例阳性。其中另2例患者分别在治疗第58天和87天时融合基因转阴。3个月时总CMR率为66.7％，6例达CMR患者中BCR-ABL融合基因P190类型3例，P210类型3例。12例患者中1例接受allo-HSCT，其余均继续化疗中。

1例达沙替尼治疗不耐受患者在服用氟马替尼治疗过程中融合基因持续阴性状态。

2例处于CR但BCR-ABL融合基因进行性升高的患者，1例在服药12 d时BCR-ABL融合基因水平下降大于1个数量级（但该患者治疗2个月时融合基因水平再次升高）；另1例患者在服药44 d时BCR-ABL融合基因水平处于稳定状态。

3. 不良反应与并发症：①血液学不良反应：9例初治和复发患者，在诱导治疗过程中，均出现Ⅳ度骨髓抑制，其粒细胞缺乏（粒缺）中位持续时间8（5～17）d，另3例患者的骨髓抑制程度为Ⅰ～Ⅱ度。②非血液学不良反应：12例患者在治疗过程中，2例出现转氨酶升高，1例出现一过性腹泻。未见皮疹、水肿及肢体疼痛等不良反应。③感染：12例患者中7例（58.3％）出现粒缺伴发热，但3例（25％）出现感染，其中肺部感染2例，单纯血流感染1例。所有患者的不良反应及感染经对症治疗后均控制。

4. 随访：所有患者接受氟马替尼治疗后的中位随访时间为139（41～254）d，中位的PFS和OS时间均未达到，在随访期间，1例患者疾病进展（融合基因再次增高），无死亡及失访病例。

## 讨论

成人Ph^+^ALL传统化疗效果差，多药化疗的5年OS率低于20％[5]。TKI应用于一线治疗后，Ph^+^ALL患者的预后有了显著的改善[6]–[8]。三代TKI药物普那替尼联合化疗的3年OS率约75％[9]，但尚未在中国上市，且价格昂贵，限制了其在临床的应用。前期TKI治疗失败的患者疗效非常差，且单独使用TKI的治疗反应也是短暂的[10]。

Ph^+^ALL复发时，选择可替代的TKI联合化疗或者嵌合抗原受体修饰T细胞（CAR-T）治疗来实现CR，后行allo-HSCT来实现长生存。在临床诊疗过程中，不管是初治还是复发Ph^+^ALL患者，我们选择TKI的种类，除了基于ABL激酶区突变位点选择敏感的TKI外，还要结合患者的年龄、身体状况、基础疾病、合并用药、药物的不良反应及价格等综合考虑。

虽然伊马替尼、达沙替尼和尼洛替尼给Ph^+^ALL患者带来了令人鼓舞的治疗效果，但长期应用后患者出现的肾功能损伤、胸腔积液和心血管事件等不良反应逐渐增多。因此，亟需寻找一种既安全又高效的TKI药物。

甲磺酸氟马替尼为一种新的口服TKI药物，在分子结构上，用吡啶环取代苯环，并导入三氟甲基，从而增强氟马替尼与BCR-ABL激酶结合的稳定性和结合力。然而，氟马替尼对c-Kit和PDGFR激酶活性的抑制作用弱于伊马替尼，对EGFR、VEGFR、c-Src和HER2激酶的磷酸化无影响[11]。另外，由于三氟甲基深入疏水口袋的程度高于尼洛替尼，故氟马替尼显示出比尼洛替尼更强的抗BCR-ABL激酶的能力[11]–[12]。此外，氟马替尼对ABL激酶ATP结合区的突变（V299L、F317L和F317I），在体外显示出更强的抑制作用[12]。但是，氟马替尼是否对T315I突变有抑制作用，目前尚未见相关报道。

在中国，氟马替尼在CML慢性期（CP）中的随机、开放、多中心Ⅲ期临床试验[13]结果显示：CML-CP患者对氟马替尼有更高、更快、更深的应答率；同时，接受氟马替尼治疗的患者，其不良事件的发生率更低。

目前，尚未检索到氟马替尼治疗Ph^+^ALL的相关报道，本研究入组12例患者，9例初治和复发患者的28 d CR率为100％（例5复发后伴T315I突变，28 d达CR），28 d CMR率为44.4％，MMR率为22.2％，治疗3个月内总的CMR率66.7％，与尼洛替尼联合多药化疗治疗初治Ph^+^ALL结果相一致[8]。1例对达沙替尼不耐受患者服用氟马替尼治疗过程中BCR-ABL融合基因持续处于阴性状态；2例患者处于CR但融合基因进行性升高，治疗中1例患者融合基因稳定，另1例患者融合基因前期出现明显下降，但治疗2个月时融合基因再次升高。12例患者治疗中未见明显不耐受现象。

综上所述，氟马替尼联合化疗治疗Ph^+^ALL疗效确切，且耐受性良好，可以作为Ph^+^ALL患者的TKI替代选择。本研究入组例数较少，随访时间较短，确切结论尚需大样本的前瞻性研究进一步证实；并且，由于例数较少，未能对初治和复发/难治患者进行亚组分析，其在初治和复发/难治患者中的疗效差异需扩大样本量观察随访。
